# Randomized multicenter trial to assess posterior capsule opacification and glistenings in two hydrophobic acrylic intraocular lenses

**DOI:** 10.1038/s41598-023-29855-8

**Published:** 2023-02-17

**Authors:** Gerd U. Auffarth, Antoine Brézin, François Lignereux, Ramin Khoramnia, Timur M. Yildirim, Thomas Kohnen, Jessica Bianco

**Affiliations:** 1 University of Heidelberg, Heidelberg, Germany; 2 Cochin Hospital, Paris, France; 3 Institut Ophtalmologique-Sourdille Atlantique, Saint-Herblain, France; 4 Goethe University, Frankfurt Am Main, Germany; 5 HOYA Surgical Optics, GmbH, Frankfurt Am Main, Germany

**Keywords:** Health care, Medical research, Optics and photonics

## Abstract

To evaluate the long-term posterior capsule opacification (PCO) formation, and glistening rate of the HOYA Vivinex (XY1) IOL compared to Alcon AcrySof (SN60WF). In this prospective, multicentric, randomized, paired-eye, open-label study, we included 87 subjects that underwent cataract surgery with IOL implantation, with 67 patients completing the 3-year follow-up. The completer population consisted of 32 subjects implanted with XY1 and 35 implanted with SN60WF. Primary endpoints consisted of the evaluation of glistenings and measurement of PCO. Secondary outcomes included Best Corrected Distance Visual Acuity (BCVA), Contrast Acuity (CA), uncorrected visual acuities, subjective refraction, medical and lens complication rates, adverse events, and optical/visual symptoms. Follow-up visits occurred at 6-months, 1-, 2- and 3-years. At 3-years follow-up, mean PCO score was 0.121 ± 0.193 for eyes implanted with Vivinex versus 0.239 ± 0.463 for AcrySof (*p* = 0.026). The Vivinex IOL showed statistically significantly lower glistening occurrence through 3-years postoperatively (0.14 ± 0.26) compared to AcrySof (1.79 ± 1.43; *p* < 0.0001). Postoperative visual acuities improved from baseline in both IOL groups (*p* < 0.0001), and remained stable through the 3-year follow-up period. Eyes implanted with a HOYA Vivinex IOL exhibited significantly lower occurrence of glistening at 3-years versus Alcon AcrySof (*p* < 0.0001). Incidence of PCO was very low and comparable in both Vivinex and AcrySof eyes.

## Introduction

Cataract is one of the leading causes of blindness and the second leading cause of moderate-to-severe vision impairment worldwide^1^. Currently available monofocal intraocular lenses (IOLs) have evolved to restore vision after removal of cataractous crystalline lenses through small-incision cataract surgery. The most common reason for a postoperative decrease in visual function is posterior capsule opacification (PCO), which is characterized by migration and proliferation of lens epithelial cells (LECs) at the posterior side of the implanted IOL⁠^1–3^. Treatment of PCO by neodymium–yttrium aluminum garnet (Nd:YAG) laser capsulotomy is effective, however, this treatment is not always considered optimal as it may lead to further complications such as increased intraocular pressure, ocular inflammation, cystoid macular edema, retinal detachment, and in rare circumstances, surgical removal of the IOL^4–6^. Therefore, much effort has been invested in optimizing IOL materials and designs to reduce PCO.

Glistenings are small (1–33 µm) fluid-filled microvacuoles that appear in IOLs when exposed to an aqueous environment as a result of phase separation caused by water developing in microvoids due to temperature fluctuations^7–9^.⁠ The formation of glistening has been reported with almost all IOLs made of polymethylmethacrylate (PMMA), silicon, hydrophilic acrylate, and most frequently with IOLs made of hydrophobic acrylic materials. Glistenings appear as small reflections of light during slit-lamp examination, and they vary in size and density^10,11^. It has been demonstrated that glistenings lead to an increase in light scattering, which can cause straylight elevation that is proportionally associated with the total number of glistenings and surface portion^12^. Previous laboratory studies suggested that IOLs made from AcrySof® material have an increased tendency towards developing glistenings compared to lenses made of other hydrophobic IOL materials. The Vivinex™ lenses, on the other hand, were observed in the laboratory to have a low tendency towards developing this material change. While the chemistry underlying these different results remains unexplained, the lens's material composition and manufacturing process are considered important contributing factors^12,13^.

The purpose of this multicentric clinical trial was to evaluate the clinical acceptability, long-term PCO formation, and the glistening rate of the HOYA Vivinex™ XY1 IOL (HOYA Surgical Optics, Tokyo, Japan) compared to the Alcon AcrySof® IOL (Alcon Laboratories, Fort Worth, Texas, USA), within a follow-up period of 3 years.

The study hypothesis proposed that the HOYA Vivinex™ XY1 IOL, compared to the AcrySof® IOL, would demonstrate a reduction in glistening, as well as comparable PCO rates, visual acuity (VA), and contrast sensitivity.

## Materials and methods

### Study design

This post-market clinical investigation was a prospective, multicentric, randomized, bilateral, comparative, paired-eye, open-label study at 4 study sites in France and Germany with a 3-year postoperative follow-up.

The Ethics Committee of the Heidelberg University, Heidelberg, Germany approved the study and informed consent was obtained from all participants. This study was conducted in accordance with Good Clinical Practices, ISO 14155:2011, the Declaration of Helsinki and all other applicable laws and regulations in Germany and France. This study has been registered in the German Clinical Trials Register on 18/08/2017 under the trial registration number DRKS00012768.

The primary endpoints were evaluation of glistening and measurement of PCO at 3-years postoperatively.

Standardized retroillumination photographs of the pseudophakic anterior segments were obtained following pupil dilation at each postoperative visit. Density areas were identified and marked on the computer screen by a grader. The individual PCO score for each eye was calculated via Evaluation of Posterior Capsule Opacification (EPCO) by multiplying the density of the opacification (graded from 0 to 4) by the fractional PCO area involved behind the IOL optic. The EPCO assessment has been performed by an external independent blinded Reading Center. EPCO calculated the surface density of opacification mathematically by performing pixel counts. The density of the opacification behind the IOL was clinically graded as follows: 0 = none; 1 = minimal; 2 = mild; 3 = moderate; 4 = severe. Additionally, routine slit lamp examinations were done to subjectively assess the degree of posterior capsular bag opacification.

At each center, a single investigator assessed the number of glistenings on the slit lamp under pupil dilatation for paired eyes. The observer was masked for the visual testing results and IOL type, and rated glistenings based on the modified severity rating scale as published by Christiansen et al*.* 2001^14^, where 0 = “None”, + 0.5 = ”rare” (< 10 glistenings), + 1 = (10 to 20 glistenings), + 2 = (20–30 glistenings), + 3 = (30–40 glistenings), and + 4 = (> 40 glistenings). The amount of glistenings were evaluated under a slit lamp field of maximum height (e.g., 10.0 mm) and 2.0 mm width.

Secondary endpoints included Best Corrected Distance Visual Acuity (BCVA), proportion of subjects achieving a BCVA in Snellen of ≥ 20/40 (0.3 logMAR), and contrast acuity (CA). Early Treatment of Diabetic Retinopathy Study (ETDRS) visual acuity charts were used in an illuminated light box to determine uncorrected visual acuity, BCVA, and low contrast VA based on the number of letters read at 4.0 m distance under photopic (85 cd/m^2^ ± 10) lighting conditions. In addition, the low contrast acuity was measured with best distance correction in place using the 10% contrast ETDRS charts at a distance of 4 m under photopic (85 cd/m^2^ ± 10) lighting conditions. Subjective refraction was also collected during study specific examinations. Postoperative examination was done using the ETDRS charts at 4 m distance under photopic lighting conditions. Safety endpoints included medical and lens complication rates, adverse events, and optical/visual symptoms.

### Randomization

Subjects had their preoperative examinations at Visit 1 and were assessed for eligibility. Randomization and the first eye surgery occurred at Visit 2 between 0 and 30 days following Visit 1. Randomization occurred prior to the procedure with a 1:1 ratio to receive either a Vivinex™ IOL or the AcrySof® IOL in the first eye operated; while the second eye received the IOL which was not implanted in the first. The second eye surgery occurred at Visit 3 between 7 and 45 days after the first eye surgery. Computer-generated randomization lists were used. A separate randomization list was prepared for each clinical site and provided in envelopes by the Sponsor. The study was organized such that all clinical personnel performing examination tests would not be informed of the randomization assignment. All sites received envelopes containing a letter indicating the name of the IOL that should be implanted; HOYA Vivinex™ or Alcon AcrySof®. Subjects had their post-operative assessments at Visit 4 where both eyes were evaluated. This visit was scheduled at 6 months (± 15 days after the second implant). The remaining post-operative visits occurred at Year 1 (12 months ± 30 days after the second implant), Year 2 (24 months ± 45 days after the second implant) and Year 3 (36 months ± 60 days after the second implant).

### Study subjects

Eighty-seven (87) subjects were enrolled in the study from normal cataract populations at the involved sites. All subjects required bilateral cataract extraction by phacoemulsification. All study subjects had to meet the following inclusion criteria: minimum 18 years of age, both male and female, bilateral clinically significant cataracts for which phacoemulsification extraction and posterior chamber IOL implantation have been planned, BCDVA projected to be better than 20/40 (0.5 decimal) following cataract removal and IOL implantation, clear intraocular media other than cataract and signed informed consent. Exclusion criteria consisted of: the need for an IOL outside the commercially available spherical power range, presence of any pupil abnormalities (non-reactive, fixed pupils, or abnormally shaped pupils), previous corneal or intraocular surgery, present corneal or intraocular pathology including pseudo-exfoliation, inability to achieve keratometric stability for contact lens wearers, anticipated intraoperative complications which may lead to IOL decentration, use of systemic or ocular medications, acute, chronic, or uncontrolled systemic disease or illness which at the surgeon’s discretion would increase the operative risk or confound the outcome of the study, pregnancy, breastfeeding or another condition associated with the fluctuation of hormones that could lead to refractive changes, concurrent participation, or participation during 30 days prior to the preoperative visit in any other clinical trial.

### Study lenses

HOYA Vivinex™ IOL (model XY1) manufactured by HOYA Surgical Optics, Tokyo, Japan is a foldable, single-piece lens for posterior chamber implantation, preloaded in a single-use injector (Vivinex™ iSert®) that automatically folds and injects the IOL into the eye. After injection, the IOL unfolds within the capsular bag. The lens is made from an ultraviolet-absorbing, high-refractive index, hydrophobic, soft acrylic polymer. The yellow Vivinex™ (XY1) IOL contains a blue-light filter in addition to the UV-light filter.

Alcon AcrySof® IOL (model SN60WF) manufactured by Alcon Laboratories, Fort Worth, Texas, USA is a single-piece, foldable, acrylic IOL which also has a blue-light filtering chromophore in addition to standard UV-light filter. The Alcon AcrySof® lens has a biconvex optic with supporting haptics and is intended for implantation in the capsular bag.

### Sample size calculation

Sample size calculation was based on the study published by Leydolt et al.^15^. As *per* standard deviations published for the AcrySof® lens, the minimum sample size required for this study was calculated at 50 subjects, using the standard deviation of 1.5. This was to demonstrate a non-inferiority margin with 80% power at an α of 0.05, if the true mean difference in PCO score between the lenses is 0. With an assumed dropout rate of 40%, the required enrolments were 84 and 97 for the standard deviations of “1.4” and “1.5”, respectively.

### Preoperative assessment

Preoperative tests were performed for each eye. Potential Visual Acuity was assessed, and the surgeon estimated the patient’s potential acuity. The patient was expected to be capable of achieving better than 20/40 (0.5) (Snellen) best corrected distance vision after cataract extraction and IOL implantation. Monocular uncorrected distance visual acuity (UDVA) was measured using a standard acuity chart. Preoperative manifest refraction was required. BCDVA was measured using a standard acuity chart. Patients required having clinically significant bilateral cataracts in both eyes. Both photopic undilated and pharmacologically dilated pupil size were measured to the nearest one-half millimetre using a pupillometer, pupil gauge card or millimetre rule.

Contact lenses were not to be worn for at least one month prior to the preoperative visit for PMMA lenses, two weeks for gas permeable lenses, and one week for extended-wear and daily-wear soft contact lenses. Corneal stability was verified for any subject who wore PMMA lenses within five years or any other type of contact lenses within six months prior to the preoperative visit. To verify stability, the keratometric measurements were repeated at least one week after the initial preoperative baseline keratometric measurement. Corneal curvature was considered stable if the difference in keratometric cylinder (vertical versus horizontal keratometric readings) between the two time points was no more than 0.50 diopter. Additionally, the difference between the two horizontal readings as well as the difference between the two vertical readings should have been no more than 0.50 diopter. Changes in keratometric axis was no more than ± 15°. Axial length was taken to determine the appropriate spherical IOL power using an A-constant. The spherical IOL power was calculated according to the patients preferred target refraction using optimized constants when available, and an appropriate IOL power calculation formula depending on the axial length.

No additional refractive procedure was performed on any of the study eyes at the time of the initial surgeries or during the postoperative study period (e.g., limbal relaxing incision (LRI), opposite clear corneal incisions (OCCI), photorefractive keratectomy (PRK) or laser-assisted in situ keratomileusis (LASIK)).

### Surgical procedure

Phacoemulsification cataract extraction surgical technique was performed. The surgeon used his or her standard small incision and decided the appropriate incision location.

The anterior capsulotomy was a continuous curvilinear capsulorhexis sized to overlap the anterior surface of the IOL (approximately 5.0–5.5 mm). After hydrodissection and hydrodeleniation phacoemulsification of the lens nucleus was performed according to the individual techniques of the individual surgeon. After cortical clean up with irrigation/aspiration the capsular bag was filled with ophthalmic viscoelastic devices (OVD). Through a 2.2 mm clear corneal incision, the IOL was placed completely within the capsular bag. A suture-less incision technique was used.

After the IOL was properly placed in the bag, the surgeon’s usual procedures for aspiration of OVD and completion of the procedure was followed. This study did not require a specific operative or perioperative medication regimen, however, a routine medication regimen form was completed by each investigator for subjects in all study groups. The recommended peri-operative medication regime included a topical antibiotic/steroid combination to control inflammation: 4 × daily 1 day preop, operative day and for 7 days post op, followed by, steroid tapering regimen 3 × daily for 1 week, followed by, 2 × daily for 1 week, followed by, once/daily for one week and then discontinued.

### Statistical analysis

Descriptive summary statistics for continuous variables included the number of observations (n), mean, standard deviation (SD), median and range (minimum, maximum) by collection timepoint and device arm (HOYA and Alcon). For categorical variables, frequency and percentage were computed. A t-test was used for continuous endpoints and p-values reported, where applicable. All statistical analyses were conducted at a 2-sided alpha level of 0.05, and confidence intervals (CI) was calculated at 95%, 2-sided.

For the PCO evaluation, the analysis was based on the paired differences of PCO scores (HOYA minus Alcon). The differences between paired observations were assumed to be normally distributed and a t-test was used for analysis. A two-sided 95% CI for the mean of the difference between the two IOLs was computed as well as the corresponding p-value. If the CI lay completely within -0.5 and 0.5, it was concluded that the HOYA IOL was comparable to the Alcon IOL. For the measurements of PCO obtained from PCO photos, EPCO was analyzed for all post-operative visits, leading to the 36-months results. For the glistening evaluation, a t-test was used on the paired differences of glistening scores (HOYA minus Alcon). The p-value for this test and the corresponding 95% CI was computed. If the p-value was < 0.05, and the mean of paired differences was less than zero, the HOYA IOL was considered superior to the control.

The statistical analysis was conducted using SAS versions 9.3 and 9.4.

## Results

### Baseline patient characteristics

The Intent-to-Treat Population (ITT) consisted of 87 subjects. As per randomization, the HOYA Vivinex™ IOL was implanted into the first eye of 43 subjects, and the Alcon AcrySof® IOL into the first eye of 44 subjects. Sixty-seven (67) subjects (the Completer population) finished the 3-year follow-up. The Completer population consisted of 32 subjects implanted with the HOYA IOL in the first eye and 35 implanted with the Alcon IOL. The remaining 20 subjects had withdrawn from the study. Demographics and baseline characteristics of the ITT are presented in Table 1. A summary of the reasons for subject withdrawal is given in Table 2. The mean age was similar in both randomization groups. The mean age of all subjects was 73.3 ± 7.8 years, with a range from 43 to 89 years. Majority of subjects were female (N = 47, 54.0%).

**Table 1:** Summary of subject demographics and baseline characteristics intent-to-treat population.

Category	HOYA-Alcon (N = 43)	Alcon-HOYA (N = 44)	Overall (N = 87)
Age(years)^[1]^, n	43	44	87
Mean (standard deviation)	72.9 (8.17)	73.7 (7.48)	73.3 (7.79)
Median	75.0	74.0	75.0
Minimum–maximum	43–82	57–89	43–89
Gender, n (%)			
Female	25 (58.1)	22 (50.0)	47 (54.0)
Male	18 (41.9)	22 (50.0)	40 (46.0)
Systemic disorder, n (%)			
Asthma	3 (7.0)	0 (0)	3 (3.4)
Atrial fibrillation	1 (2.3)	4 (9.1)	5 (5.7)
Chronic obstructive pulmonary disease	1 (2.3)	0 (0)	1 (1.2)
Coronary artery disease	2 (4.7)	3 (6.8)	5 (5.7)
Diabetes mellitus	5 (11.2)	4 (9.1)	9 (10.4)
Heart failure	0 (0)	1 (2.3)	1 (1.2)
Dyslipidemia	11 (25.6)	10 (22.7)	21 (24.1)
Hypertension	25 (58.1)	23 (52.3)	48 (55.2)
Hypothyreosis	5 (11.6)	5 (11.4)	10 (11.5)
Designated first operative eye			
Left eye	18 (41.9)	17 (38.6)	35 (40.2)
Right eye	25 (58.1)	27 (61.4)	52 (59.8)
Under the opinion of the Investigator, the eyes will achieve a best corrected distance vision of 20/30 after cataract surgery			
Left eye			
Yes	43 (100.0)	44 (100.0)	87 (100.0)
No	0 (0.0)	0 (0.0)	0 (0.0)
Right eye			
Yes	43 (100.0)	44 (100.0)	87 (100.0)
No	0 (0.0)	0 (0.0)	0 (0.0)

Percentages are based on the number of subjects in the sequence.

[1] Age is the number of full years from date of birth to the date of informed consent.

**Table 2:** Subject disposition (all randomized subjects).

Category	HOYA-Alcon (N = 43)		Alcon-HOYA (N = 44)		Overall (N = 87)	
	n	(%)	n	(%)	n	(%)
Intent-to-treat population	43	(100.0)	44	(100.0)	87	(100.0)
Completer population	32	(74.4)	35	(79.5)	67	(77.0)
Per-protocol population	5	(11.6)	9	(20.5)	14	(16.1)
Withdrew from the study	11	(25.6)	9	(20.5)	20	(23.0)
Reason for early withdrawal adverse event	2	(4.7)	2	(4.5)	4	(4.6)
Voluntary withdrawal	3	(7.0)	2	(4.5)	5	(5.7)
Lost to follow-up	1	(2.3)	3	(6.8)	4	(4.6)
Physician’s decision	2	(4.7)	0	(0.0)	2	(2.3)
Death	3	(7.0)	1	(2.3)	4	(4.6)
Other	0	(0.0)	1	(2.3)	1	(1.1)

Percentages are based on the number of randomized subjects in the sequence.

The intent-to-treat population consists of all randomized subjects who have at least one IOL implanted.

The completer population consists of all the ITT subjects who have both IOLs implanted with follow-up through the primary assessment time point.

The per protocol population includes all subjects who are in the completer population with no significant protocol deviations.

### Posterior capsule opacification (PCO) evaluation

The EPCO analysis was performed in paired eyes as described in the material and methods. For this analysis, based on picture quality, there were 57 patients available with paired data at 3-years of follow up. The mean PCO score of the EPCO analysis was 0.121 ± 0.193 (range: 0.000 to 0.718) for eyes implanted with the Vivinex™ IOL, and 0.239 ± 0.463 (range: 0.000 to 2.564) for eyes implanted with the AcrySof® IOL. The results of the t-test showed that there was a statistically significant difference between the IOL groups (*p* = 0.026). The 95% confidence interval concluded that PCO was comparable for both IOLs.

**Figure 1:**
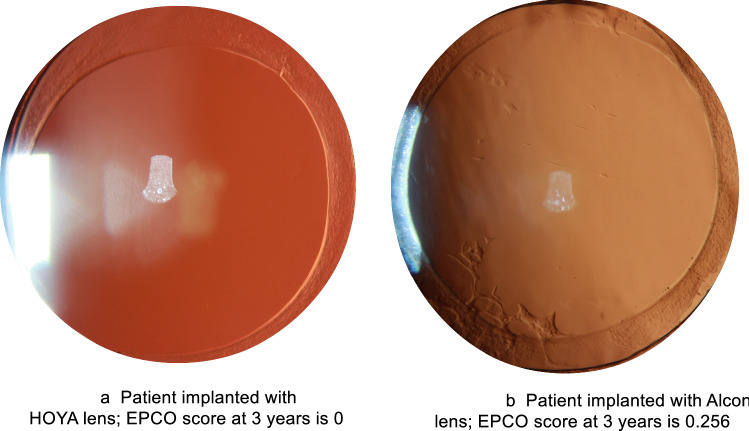
Patient implanted with a Vivinex lens; EPCO score at 3 years is 0 (**a**) and a patient implanted with Acrysof lens; EPCO score at 3 years is 0.256 (**b**).

There were 44 patients out of 57 (77.19%) implanted with the HOYA IOL that displayed an EPCO score from > 0 to 1 (none to minimal), and 42 patients out of 57 (73.68%) implanted with the Alcon IOL that displayed an EPCO score from > 0 to 1 (none to minimal). There were 13 patients (22.81%) implanted with the Vivinex™ IOL and 13 patients (22.81%) implanted with the AcrySof® IOL that did not display any signs of PCO. It should be noted that no patient implanted with the HOYA IOL reported mild, moderate, or severe PCO; 2 patients (3.51%) implanted with the Alcon IOL reported minimal to mild PCO, and mild to moderate PCO. Figure 1 reports the comparison of the EPCO scores between the Vivinex™ and AcrySof® IOLs.

For the assessment of subjective PCO, there were 67 patients with available data from paired eyes. The subjectively evaluated mean PCO score was 0.299 ± 0.551 (range: 0.000–2.000) for eyes implanted with Vivinex™, and 0.478 ± 0.841 (range: 0.000–4.000) for eyes implanted with AcrySof®. The result of the t-test showed a statistically significant difference between the IOL groups (*p* = 0.045). The 95% confidence interval concluded that PCO was comparable between the IOLs. At 3-years, overall, 74.63% of eyes implanted with a Vivinex™ IOL did not show any PCO versus 68.66% of eyes implanted with AcrySof®. The percentages of trace PCO were comparable between the two groups. However, eyes implanted with an AcrySof® IOL had more reports of mild PCO. Additionally, the scores of moderate and severe PCO were only reported in eyes implanted with an AcrySof® IOL. Results are summarized in Table 3.

**Table 3:** Frequency and proportion of subjective posterior capsule opacification (PCO) at 3-years.

PCO severity	Number of patients and percentage (%) implanted with Alcon IOL	Number of patients and percentage (%) implanted with HOYA IOL
Score 0 (none)	46 (68.66%)	50 (74.63%)
Score > 0–1 (none to minimal)	13 (19.40%)	14 (20.90%)
Score > 1–2 (minimal to mild)	6 (8.96%)	3 (4.48%)
Score > 2–3 (mild to moderate)	1 (1.49%)	0 (0.00%)
Score > 3–4 (moderate to severe)	1 (1.49%)	0 (0.00%)

*IOL* intraocular lens.

The results from the subjective evaluation of PCO as well as from the EPCO analysis confirm a comparable performance between eyes implanted with Vivinex™ and AcrySof® IOLs.

Out of 67 patients, there were no Nd:YAG laser capsulotomies performed up to 3-years postoperatively in eyes implanted with the HOYA IOL. For eyes implanted with the Alcon IOL, one capsulotomy was performed (1.5%). This capsulotomy was done within the first 6-months after implantation. This result demonstrated low occurrence of PCO in both IOL groups and comparable performance regarding frequency of YAG laser capsulotomies.

### Glistenings

Glistenings were assessed at 3-years in 67 patients. The HOYA lens showed a trend of low glistening occurrence through 3-years postoperatively (0.14 ± 0.26; range: 0.0–1.0).

Compared to the Vivinex™ IOL, the mean glistening score of the AcrySof® IOL at 3-years was statistically significantly higher at each postoperative visit, 1.79 ± 1.43, *p* < 0.0001.

Within the HOYA group, the overall glistening scores remained low, with only 2 subjects (2.99%) observing a glistening score of + 1. Up to 36-months, there were no cases of mild to severe glistening with any Vivinex™ IOL, while approximately 50% of the AcrySof® group showed glistening scores of + 2 to + 4 (Table 4).

**Table 4:** Glistening scores—frequency/proportion per visit.

Glistening scores	Number of patients and percentage (%) implanted with Alcon IOL	Number of patients and percentage (%) implanted with HOYA IOL
Score 0	8 (11.94%)	50 (74.63%)
Score 0.5	14 (20.90%)	15 (22.39%)
Score 1	12 (17.91%)	2 (2.99%)
Score 2	12 (17.91%)	0 (0.00%)
Score 3	7 (10.45%)	0 (0.00%)
Score 4	14 (20.90%)	0 (0.00%)

*IOL* intraocular lens.

### Refractive results

At baseline, the mean Uncorrected Distance Visual Acuity (UDVA) and Best-Corrected Distance Visual Acuity (BCDVA) values of the Completer population (n = 67) did not differ significantly between the eyes implanted with a Vivinex™ or AcrySof® IOL. The postoperative UDVA and BCDVA showed a statistically significant improvement from baseline in both IOL groups (*p* < 0.0001). There was no statistical difference between the IOL groups at all postoperative follow-up visits, and the postoperative outcomes of UDVA and BCDVA remained stable from 0.5-year postoperatively until the 3-year examination (Table 5).

**Table 5:** UDVA and BCDVA values of the completer population (n = 67).

	HOYA		Alcon		*t*-test (*p*-value)
UDVA					
logMAR	Mean ± stdev	Min–max	Mean ± stdev	Min–max	
Preop	0.62 ± 0.82	1.10–0.00	0.60 ± 0.78	1.10–0.00	0.641
0.5-year	0.20 ± 0.89	0.82–< 0.00	0.19 ± 0.89	0.74–< 0.00	0.705
3-years	0.19 ± 0.90	0.88–< 0.00	0.20 ± 0.87	1.04–< 0.00	0.810
BCDVA					
logMAR	Mean ± stdev	Min–max	Mean ± stdev	Min–max	
Preop	0.30 ± 0.89	0.78–< 0.00	0.30 ± 0.88	1.10–< 0.00	0.834
0.5-year	0.00 ± 0.98	0.38–< 0.00	0.00 ± 1.00	0.40–< 0.00	0.419
3-years	0.00 ± 1.00	0.32–< 0.00	0.00 ± 0.95	1.04–< 0.00	0.667

*UDVA* uncorrected distance visual acuity, *BCDVA* best-corrected distance visual acuity, *logMAR* logarithm of the minimum angle of resolution, *stdev* standard deviation.

The low contrast acuity (low CA) was also measured. The 0.5-year postoperative mean low CA values of the Completer population did not differ significantly between the eyes implanted with a Vivinex™ IOL and those implanted with an AcrySof® IOL (HOYA: 0.23 ± 0.96, range: 0.58–< 0.0; Alcon: 0.23 ± 0.98; range: 0.64 to 0.0; *p* = 0.284). The postoperative outcomes remained stable from 0.5-year postoperatively until the 3-year follow-up visit with no statistical difference between the IOL groups.

Subjective refraction was also collected during study specific examinations. Mean postoperative subjective cylinder at 0.5-year was not different between eyes implanted with a HOYA IOL (0.593 ± 0.520 D; range: 0.00 to − 2.25 D) and eyes implanted with an Alcon IOL (0.549 ± 0.528 D; range: 1.00 to − 2.00 D); *p* = 0.5229. This remained similar through the 3-year postoperative examination where mean postoperative subjective cylinder was 0.758 ± 0.542 D (range: 0.00–2.00 D) in HOYA eyes, and 0.742 ± 0.553 D (range: 0.00–2.00 D) in Alcon eyes; *p* = 0.8036.

### Safety outcomes

There were no reports of adverse events leading to lens removal or unanticipated adverse device effects in either group. Two AEs were related to the procedure in the Vivinex™ group. There were 12 ocular AEs (17.9%) in eyes implanted with a HOYA lens, with none being related to the device. For the Alcon eyes, 8 ocular AEs (11.9%) were reported with four being related to the procedure. There were total of 4 deaths in both IOL groups, which were not related to the cataract surgery or IOL implantation. Non-serious macular edema (rated mild and moderate) was reported in 2 subjects implanted with a Vivinex™ IOL (3.0%) and one subject implanted with an AcrySof® IOL (1.5%). Corneal edema of mild severity was reported in one subject with an Alcon IOL (1.5%). Mild elevation of intraocular pressure (IOP) was reported in one subject of each group (1.5%). With respect to serious adverse events, there was one report of a retinal pathology (detachment/hole/tear) in a HOYA subject (1.5%), and a haemorrhage in one subject of each group (1.5%). One secondary surgical intervention was reported in a subject implanted with Vivinex™. There were no reports of any persistent adverse events. No serious adverse device effects related to the IOLs were reported during the study.

## Discussion

The results achieved in this study aligned with the null hypothesis that the HOYA Vivinex™ IOL, when compared to the Alcon AcrySof® IOL, would demonstrate a low occurrence of glistening, with comparable PCO, visual acuity, and contrast sensitivity.

PCO is a common complication after IOL implantation, leading to decrease in visual acuity and unsatisfactory results. Although Nd:YAG laser capsulotomy is currently the state-of-the-art treatment, it may lead to further complications^6^. As a result, the need to develop an IOL material that prevents the formation of PCO is seen throughout the industry. Many studies have also expressed the importance of the posterior edge as a major influence on preventing PCO, acting as a barrier to inhibit LECs from migrating beneath the IOL optic^6,16,17^.⁠ With respect to Vivinex™ IOLs, a surface treatment using ultraviolet/ozone (UV/O_3_) was developed and patented by HOYA to modify the IOL, aiming to enhance adhesion between the IOL and the posterior capsular bag. This tight adhesion makes it particularly difficult for any residual LECs to proliferate in this area and cause PCO. Likewise, the Vivinex™ IOL is a biconvex lens with aspheric design and squared textured (posterior) optic edge to minimize PCO.

The results from the EPCO analysis and the subjective evaluation of PCO showed a statistically significant difference in favour of eyes implanted with a Vivinex™ IOL versus AcrySof®. Design of an IOL plays an important role in the prevention of PCO formation. An IOL with a square posterior optic edge may prevent the migration of LECs under the optic of the IOL^18,19^. Both the Vivinex™ and AcrySof® IOLs have a square posterior optic edge design.

A previous study published by Leydolt et al.^6^ compared Vivinex™ XY1 with AcrySof® SN60WF to identify any resulting differences in PCO development between the two IOLs. The results of this study showed that Vivinex™ XY1 displayed significantly lower objective PCO rates compared to AcrySof® SN60WF^6^. In the same study, though not statistically significant, Nd:YAG rates were also lower in the Vivinex™ XY1 group^6^. These results are in line with the results of the current study, since no Nd:YAG laser capsulotomy was performed in any eyes implanted with a Vivinex™ IOL.

The clinical significance of glistenings is a subject of debate, with some studies showing an influence of glistening on contrast sensitivity^20,21^ and visual acuity^14^, while others have indicated that a limited number of glistenings had no effect on image quality or visual function^22,23^.

Recent studies on accelerated aging of hydrophobic intraocular lenses have shown that the current AcrySof® material (in contrast to the new Clareon Material of the same manufacturer) has significantly higher formation of glistenings compared to other marketed hydrophobic IOLs^24–26^. The main clinical problem with glistenings is not reduction of visual acuity but a reduction of visual quality by high levels of stray-light^12,27–29^. These straylight levels can be comparable with straylight created by a cataract of a 70-year-old natural crystalline lens^27^.

The presence of glistenings has been shown to be related to the manufacturing process and the material an IOL is made from^30^. When an IOL is molded instead of lathe cut, the process allows gaps or vacuoles to form within the optic material, which then allow water to collect within the IOL, even after successful implantation. The resultant glistenings cause light to scatter as it enters the eye, which can result in reduced contrast sensitivity^31^. Glistenings develop over time, indicating that long-term outcomes are not fully known, and although some cases have resulted in IOL explantation, this is considered rare^32^. Vivinex™ lenses are made with 2-[4-(2-hydroxyethoxy)-phe-noxy]-ethyl acrylate (HPEA), for high refractive index and glistening inhibition. As demonstrated in this study, glistening was very rare in Vivinex™ IOLs and the result was statistically significantly lower than with AcrySof® at each postoperative time point. This finding accords with laboratory studies on lenses made of these materials^13^.

The study also presented successful restoration of visual acuity. The mean BCDVA under photopic lighting conditions was comparable in both IOL groups at all time points. The results are consistent with the results reported in the study by Lundström et al.^33^.

Low contrast acuity did not differ significantly between the IOL groups and outcomes remained stable over time at the level of acceptable functional vision.

Complications and adverse events observed were comparable between the IOL groups. There were no persistent ocular adverse events. All adverse events and complications that occurred were known for patients undergoing cataract surgery with IOL implantation, and no new risks were identified.

One limitation of this study is that for the EPCO results, there were only 57 patients available with paired data at 3-years of follow-up as 20 patients withdrew from the study. However, it should be noted that the analysis has been conducted within the minimum sample size as based on the study published by Leydolt et al.^15^. Another limitation is the excellent PCO preventive effect of both IOLs leading to average EPCO scores of only 0.12 and 0.2, respectively on a scale up to 4.0. Having these small amounts of opacified area made it difficult to show highly significant differences. Regarding the evaluation of glistenings, another limitation of the study is that we cannot rule out that there would be a minimal amount of discrepancy from multiple blinded examiners following the same protocol. Even though a follow-up period of 3 years revealed differences in the number of glistenings between the two investigated IOL materials, longer follow-up periods must be considered, in order to be able to draw conclusions about the long-term glistening development in these IOLs. In conclusion, the results of this 3-year study showed comparable incidence of very low amounts of PCO in eyes implanted with both a HOYA Vivinex™ IOL and an Alcon AcrySof® IOL, resulting from the subjective evaluation of PCO and EPCO analysis. Over the study period, Nd:YAG laser capsulotomy was not required for eyes implanted with Vivinex™, but for one eye implanted with the AcrySof® IOL. At 3-years follow-up, the occurrence of glistening was significantly lower in eyes implanted with Vivinex™ versus AcrySof®.

## Data Availability

All data generated or analysed during this study are included in this published article.
